# Identification of streptococcal small RNAs that are putative targets of RNase III through bioinformatics analysis of RNA sequencing data

**DOI:** 10.1186/s12859-017-1897-0

**Published:** 2017-12-28

**Authors:** Ethan C. Rath, Stephanie Pitman, Kyu Hong Cho, Yongsheng Bai

**Affiliations:** 1 Department of Biology, Indiana State University, Terre Haute, IN 47809 USA; 2 The Center for Genomic Advocacy, Indiana State University, Terre Haute, IN 47809 USA

**Keywords:** *Streptococcus pyogenes*, Small RNAs, RNase III, RNA sequencing, Bioinformatics

## Abstract

**Background:**

Small noncoding regulatory RNAs (sRNAs) are post-transcriptional regulators, regulating mRNAs, proteins, and DNA in bacteria. One class of sRNAs, *trans-*acting sRNAs, are the most abundant sRNAs transcribed from the intergenic regions (IGRs) of the bacterial genome. In *Streptococcus pyogenes*, a common and potentially deadly pathogen, many sRNAs have been identified, but only a few have been studied. The goal of this study is to identify *trans*-acting sRNAs that can be substrates of RNase III. The endoribonuclease RNase III cleaves double stranded RNAs, which can be formed during the interaction between an sRNA and target mRNAs.

**Results:**

For this study, we created an RNase III null mutant of *Streptococcus pyogenes* and its RNA sequencing (RNA-Seq) data were analyzed and compared to that of the wild-type. First, we developed a custom script that can detect intergenic regions of the *S. pyogenes* genome. A differential expression analysis with Cufflinks and Stringtie was then performed to identify the intergenic regions whose expression was influenced by the RNase III gene deletion.

**Conclusion:**

This analysis yielded 12 differentially expressed regions with >|2| fold change and *p* ≤ 0.05. Using Artemis and Bamview genome viewers, these regions were visually verified leaving 6 putative sRNAs. This study not only expanded our knowledge on novel sRNAs but would also give us new insight into sRNA degradation.

## Background

*S. pyogenes*, also known as Group A *Streptococcus* (GAS), is an important human pathogen that affects 700 million people worldwide each year resulting in about 500,000 deaths due to various complications [1]. This Gram-positive bacterium can cause a wide range of both external and internal diseases. External or superficial infections include pharyngitis, impetigo, erysipelas, vaginitis, and post-partum infections [2, 3]. Although detrimental, these diseases are not typically fatal. However, some can progress to necrotizing fasciitis and scarlet fever which are far more problematic [4]. Internally, GAS can cause necrotizing fasciitis, cellulitis, septic arthritis, puerperal sepsis, meningitis, abscess, osteomyelitis, endocarditis, and peritonitis, all which can involve toxic shock-like syndrome [5]. The type of resulting infection is determined by how the bacteria are contracted and by the serotype. Serotypes are determined by the sequence on the 5′ end of the *emm* gene, which encodes for M protein [6]. However, there are many different factors beyond M protein that affect GAS virulence [7]. Further understanding of the mechanisms behind *S. pyogenes* infections could lead to new ways of treating this pathogen. Likewise, more knowledge of the pathogenesis of this pathogen could play a role in comparatively studying similar mechanisms in other pathogens.

**Fig. 1:**
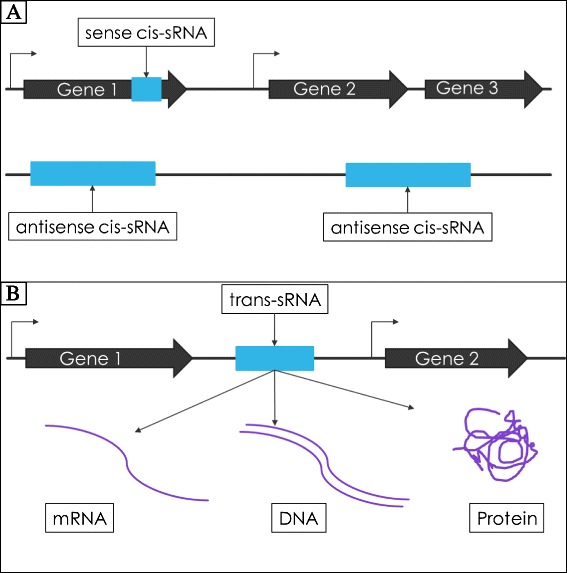
**a** Types of *cis*-sRNAs and their gene locations in the chromosome (**b**) The gene location of *trans*-sRNA and its targets. Thick black arrows represent theoretical genes and their directions, blue boxes denote theoretical locations of sRNA genes, and purple items represent targets of a *trans*-sRNA

The most ubiquitous post-transcriptional regulator across all bacteria is the small non-coding regulatory RNA (sRNA) [8]. These sRNAs are incredibly important in translational regulation by controlling mRNA activity [9]. Although less common, some sRNAs can also affect DNA and proteins [10–12]. These sRNAs come in two major types: *cis*-acting sRNAs (*cis*-sRNAs) and *trans*-acting sRNAs (*trans*-sRNAs). The defining characteristic of *cis-*RNA is that it is transcribed from the same region as its target (antisense *cis*-sRNA) or even within the same mRNA as its potential target (sense *cis*-sRNA) [13]. *Cis*-sRNA typically targets either the gene (or genes) with which it is transcribed or the gene(s) opposite to it (Fig. 1a) [13]. *Cis*-sRNAs have a much more limited scope of targets than *trans-*sRNAs, which could be any in the RNA transcriptome. As such, this paper is focused on the discovery of *trans*-sRNA.

*Trans*-sRNAs employ many different modes of action to regulate RNA expression [14]. They can repress mRNA translation, enhance mRNA translation, increase degradation of mRNA, or block degradation. Some sRNA can function in multiple manners, depending on their targets. These mechanisms have been described in detail and are usually dependent on the sRNA binding site on the target mRNA. Unlike *cis*-sRNAs, *trans*-sRNAs are transcribed from intergenic regions (IGRs) throughout the genome (Fig. 1b), making these sRNA easier to detect both computationally and through visual analysis [15]. The development of sRNA identification-related bioinformatics tools has allowed for the prediction of many novel sRNAs in a wide range of bacteria, including *S. pyogenes* [13]. Some sRNAs are involved in regulating virulence of pathogens, and to date, three *trans*-sRNAs have been identified as regulators of virulence factors in *S. pyogenes*, PelRNA, RivX, and FasX [8, 16–18]. By studying basic sRNA degradation, our research has the potential to eventually be applied to the research of the degradation of virulence factor-regulating sRNAs as well.

Currently, the degradation of sRNAs is not well known. It is thought that the degradation of sRNA is reliant on various ribonucleases similar to the process of mRNA degradation. Ribonucleases are enzymes that assist in the breakdown of RNA in cells [19]. The endoribonuclease RNase III whose substrates are double-stranded RNAs has shown great promise as a potential post-transcriptional regulator of many sRNAs [20, 21].

The RNA sequencing (RNA-seq) data processing has been simplified to an easy to use open source pipeline that has been widely used for alignment and observation of RNA-seq data. This process relies on many different algorithms that can yield multiple types of results, from simple alignments to complex differential expression analyses. This pipeline starts with Bowtie2 as a base which creates an index of the genomic file [22]. The index created here is used for alignment of the short reads by the Burrow-Wheeler transformation algorithm (BWA) based RNA alignment software TopHat [23]. After alignment, the BAM (or SAM) files are then passed to Cufflinks [24, 25]. Cufflinks processes the alignments into an assembled form. All assemblies are sent to Cuffmerge for further analysis [24, 25]. Finally, Cuffdiff compares the expression of each transcript through read depth and identifies any differentially expressed transcripts [24, 25]. We also applied a second pipeline named as the RSEM/EBSeq pipeline [26–28] for differential expression cross analysis. This pipeline requires complete annotation of unannotated regions, provided by StringTie [26]. This annotation is then used to align, assemble, calculate expression, and merge these values into a matrix using RSEM [27]. Lastly, differential expression is performed using EBSeq [28].

**Fig. 2:**
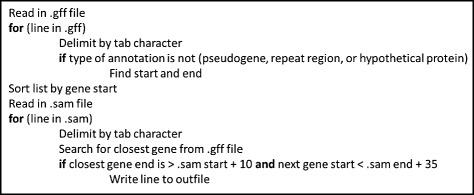
Pseudocode for intergenic region detection script

**Fig. 3:**
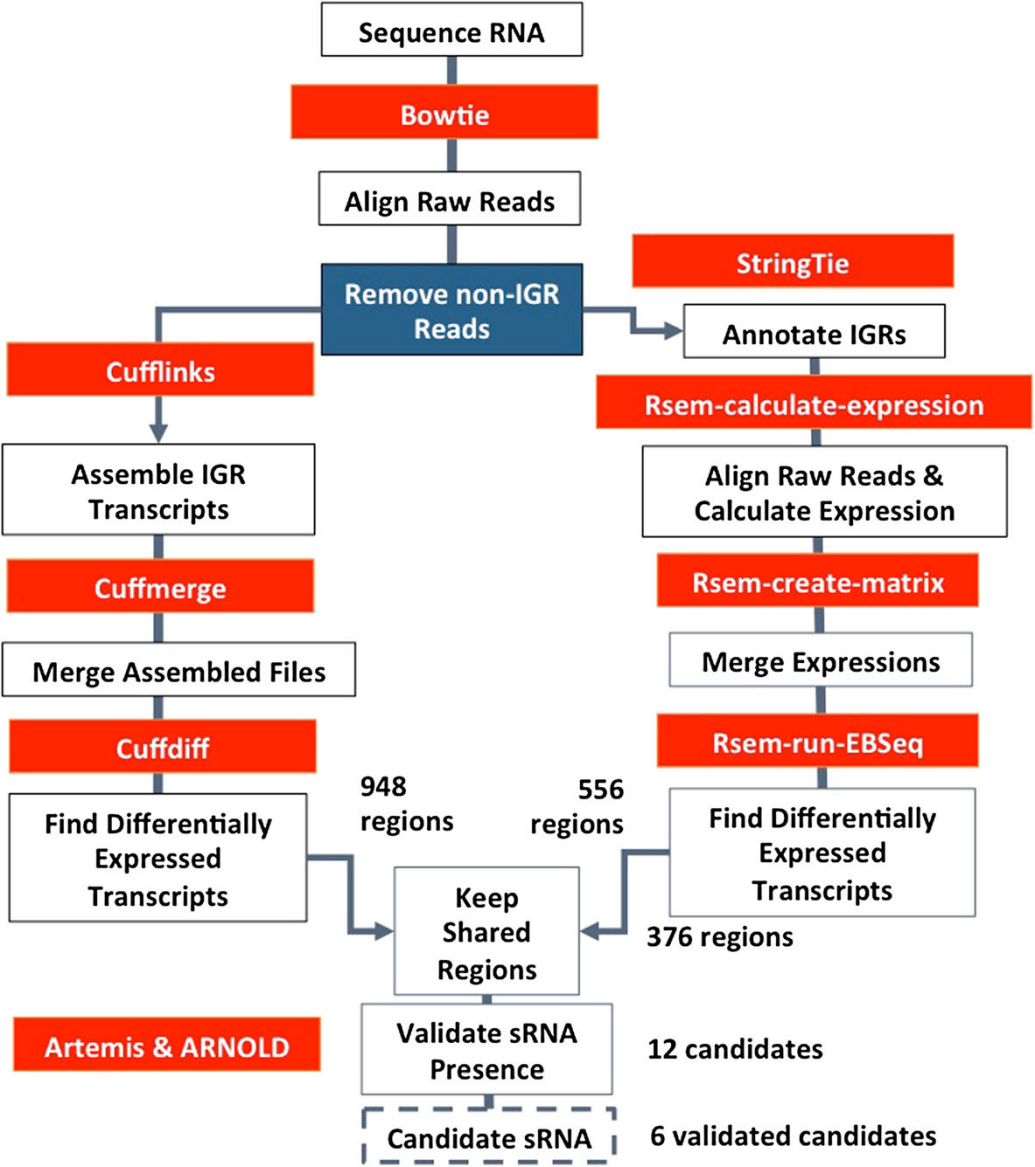
Pipeline for RNA-seq analysis for intergenic region detection. Red boxes denote software used while the solid blue box is software developed in house. This pipeline details two separate pipelines for analyzing RNA-seq data focusing on intergenic regions with measureable expression. Both pipelines were used to create a final prospective list, also denoted in this workflow

The goal of this study was to identify differentially expressed IGRs potentially affected by the endoribonuclease RNase III in *S. pyogenes* and then determine whether they are sRNAs. First, a bioinformatics approach was developed by employing two different pipelines (Figs. 2, 3) to analyze the RNA sequencing data of the wild type strain, HSC5, and an RNase III null mutant for differential expression. The IGRs reflecting >2 fold-difference were analyzed visually to determine any potential sRNAs.

## Methods

### *Streptococcus pyogenes* growth condition

*S. pyogenes* HSC5 was used for all experiments and strain construction. HSC5 is a non-mucoid M14 serotype lab strain [29], and has recently been sequenced [30].Todd Hewitt media (BBL) with 0.2% yeast extract (THY media) was used to cultivate *S. pyogenes*. For growth in liquid media, *S. pyogenes* was cultured at 37 °C in sealed tubes without shaking. To produce solid media, Bacto agar was added to a final concentration of 1.4% (wt/vol). *S. pyogenes* grown on plates (solid media) was incubated in anoxic conditions using the Gas Pak EZ anaerobe containment system (Catalogue no. 260678, BBL). *Escherichia coli* Top10 (Invitrogen) was used for plasmid construction. *E. coli* was cultured in Luria-Bertani broth (LB) at 37 °C with shaking. When appropriate, optimum concentration of antibiotics was added to the media.

### Creation of a nonpolar in-frame deletion mutant of the RNase III gene, *rnc*

An in-frame deletion allele of *rnc* was created as follows. The primers of 5outRNase3IFD-*Kpn*I (aaa**ggtacc**caaagagttagcgcatatgacg) and 3outRNase3IFD (cagtatctttagtctgtctttcttgagc) were used to amplify a 2.02 kb DNA fragment including *rnc*. This amplified fragment was digested and inserted between *Kpn*I and *Xba*I restriction sites in the multiple cloning site of pCRII (Invitrogen). The *Kpn*I restriction site is located in the primer sequence of 5outRNase3IFD-*Kpn*I, which is underlined, and the *Xba*I site is located near the 3′ end of the PCR-amplified product. The resulting plasmid was then used as a template in an ‘inside-out’ PCR reaction with the primers of 5inRNase3IFD-*Xma*I (aaa**cccggg**attagtgagaaaggacctgccc) and 3inRNase3IFD-*Xma*I (aaa**cccggg**ctctgaaataatcaattgtagaacagcg). Restriction of this fragment with *XmaI* followed by subsequent re-ligation resulted in a nonpolar inframe deletion that replaces DNA sequence encoding Y61 – V181 of RNase III with the sequence of cccggg encoding PG. The in-frame deletion allele of *rnc* was then inserted between the *Bam*HI and *Xba*I restriction site in the *S. pyogenes*-*E. coli* shuttle vector, pJRS233. The generated plasmid, pJRS233::*rnc*-IFD, was used to replace the wild type *rnc* with the in-frame-deleted *rnc* by a method that employs the temperature sensitivity of the pJRS233 replication origin [31].

### RNA extraction from *S. pyogenes*

The wild type (HSC5) and the RNase III mutant (ΔRNase III) were grown in THY media to the exponential phase (OD600 of 0.4–0.5). Then, total RNA was extracted using the combination of the miRNeasy kit (Qiagen) and the FastPrep beadbeater (MP biomedicals). An *S. pyogenes* cell pellet from 10 ml culture was resuspended in 700 ml of the Qiazol lysis reagent (Qiagen) and transferred to a Lyse Matrix B blue cap tube (MP biomedicals). Cells were then lysed by the beadbeater, FastPrep 24 (MP biomedicals) at the speed of 6.0 for 40 s twice. The remaining procedure for RNA extraction followed the manufacturer’s protocol of the miRNeasy kit. During RNA purification, RNase-Free DNase (Qiagen) was treated on column to remove residual DNA. The A260/A280 ratio of the extracted RNA was measured with Eppendorf BioSpectrometer® to determine the RNA concentration and purity (accepted if >1.8). The extracted RNA was mixed with 1 ul of RNasin (Promega Recombinant RNasin Ribonuclease Inhibitor, 40 u/ml), and treated with the RNAstable kit for safe transport.Next-generation sequencing, RNA-Seq.

The extracted RNA samples were submitted to Macrogen Corporation (Rockvill, MD, USA) for RNA-Seq assays. The quantity, integrity and purity of total RNA were assessed using Ribogreen (Life technologies, cat# R11490) and Agilent Bioanalyzer 2100. RNA was subjected to rRNA depletion using the RiboZero Meta-Bacteria kit (Epicentre Biotechnologies, Madison, WI USA, catalog # MRZMB126) and cDNA was generated from the rRNA depleted RNA using the NEBNext mRNA Sample Prep kit (New England Biolabs, Ipswich, MA USA, catalog# E6110). cDNA was profiled using Agilent Bioanalyzer, and subjected to Illumina library preparation using NEB Next reagents (New England Biolabs, Ipswich, MA USA, catalog# E6040). The quality and quantity and the size distribution of the Illumina libraries were determined using an Agilent Bioanalyzer 2100. The libraries were then submitted for Illumina HiSeq2000 sequencing according to the standard operation. Paired-end 90 or 100 nucleotide (nt) reads were generated.

### Alignment of RNA-seq data

To detect sRNAs expressed from the RNA-seq data, raw sequencing data was aligned using TopHat [23] with a Bowtie2 index file [22] to the HSC5 genome.fasta (GCF_000422045.1_ASM42204v1_genomic.fna). TopHat produced four BAM files for each condition (2 for each replicate). These were converted into SAM files using samtools [32]. A custom Python script was written to report the aligned reads that appeared in the IGRs of the HSC5 genome. This script takes in a SAM file and an annotated genome file (in .gff format) and identifies any reads that fall within an IGR. An IGR was defined as a location that was 10 bp downstream from the end of a known gene and 35 bp upstream from the next downstream gene. Any reads found within these regions were then written into a new SAM file (Fig. 2).

### Intergenic region detection

Once the reads that aligned to IGRs were detected and isolated, each SAM file was loaded into the differential expression pipeline provided by the Cufflinks package [22–25] (Fig. 2). Each SAM file was run through Cufflinks and then merged through Cuffmerge. These files were then used for a final run through Cuffdiff [22–25]. The final differential expression from the Cufflinks pipeline gave 938 potential regions.

### Alternate differential expression

In order to confirm the regions detected by Cufflinks, a secondary pipeline for differential expression analysis using RSEM was used [26–28]. RSEM requires an annotated genome in order to perform a differential expression analysis, therefore our detected regions had to be annotated. The SAM files produced by our novel script were processed through the Stringtie software to annotate the IGRs that were detected by our program [26]. The headers of these files were removed and they were concatenated into a singular .gff file. This .gff along with the raw sequence files were used for rsem-calculate-expression. The transcript files produced from the first step were then combined into an expression matrix using rsem-generate-data-matrix. Finally, this matrix was run through rsem-run-ebseq [27]. EBSeq differential expression analysis yielded 556 differentially expressed IGRs [28].

### Final candidate choice

The results of both Cufflinks differential expression and EBseq were compared to find similar regions using a custom Python script. A total of 376 regions were detected in common by both pipelines and selected for further analysis. Any statistically significant differentially expressed regions with at least a 2-fold change and a *p*-value ≤0.05 were used for further analysis.

### Visual confirmation

Using the genome viewer tools Artemis and Bamview, the statistically significant differentially expressed IGRs were analyzed for visual confirmation as sRNAs [33]. Respective regions in WT and RNase III mutant RNAseq data were compared.

### ARNold confirmation

Most sRNAs have rho-independent terminators at their 3′ ends. As such, prediction software was used to determine if these terminators were present in any of the current candidates. Using IGVs regions of interest, we compiled the sequences of each candidate IGR into a multi-fasta file. This file was uploaded into ARNold and run using default settings [34–36]. The results were then used to further classify the candidates.

## Results and Discussion

### Creation and confirmation of an RNase III gene inframe deletion mutant

The interaction between an sRNA and its mRNA target forms a double strand RNA structure that can be the substrate of RNase III. Therefore, RNase III could be an enzyme at least in part responsible for the degradation or processing of some sRNAs. To test this possibility, a deletion of the *rnc* gene encoding RNase III was performed, and the expression of putative sRNAs of the mutant was compared to that of the wild type.

There are several strategies to inactivate genes in *Streptococcus*: allelic replacement, directed insertional inactivation, and in-frame deletion. The first two methods are relatively easy to carry out. However, the mutated gene could generate an undesired expression pattern of the downstream genes in the same operon. Sequence analysis showed that immediately downstream of *rnc,* there was a gene with the same direction*,* encoding the putative chromosomal partition protein SMC. Since the distance between both genes is short, they might form an operon. For this reason, the in-frame deletion approach was used to delete *rnc*.

**Fig. 4:**
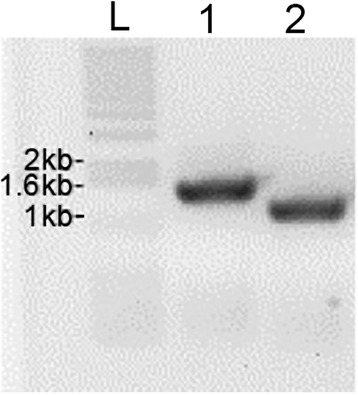
PCR confirmation of the RNase III in-frame deletion mutant. Lane L shows DNA size markers. Lane 1 shows the PCR product using genomic DNA from the wild type (HSC5) strain. The product has the expected size of ~1.2 kb. Lane 2 shows the PCR product using genomic DNA from the *rnc* inframe deletion mutant. The PCR product is supposed to be 372 bps smaller than that of the wild type. The primers used for this PCR are 5-GGTCTACTGACAAATATGAAAGGG-3 and 5-CAGTATCTTTAGTCTGTCTTTCTTGAGC-3

In the in-frame deletion method, the gene of interest is inserted into a vector whose replication is dependent on temperature. Then, a large central portion of the gene is deleted in-frame, which preserves the reading frame of the message. After inserting a DNA containing *rnc* and its flanking regions into the *E. coli – S. pyogene* shuttle vector pJRS233, we deleted 372 bp that represents 54% of the *rnc* sequence through an inverse PCR technique. We confirmed through DNA sequencing that the construct contained the deletion and still maintained the reading frame. Later, the construct was transferred to the wild type HSC5 strain, and necessary steps were performed to obtain the mutant. PCR was performed to confirm the in-frame deletion in the chromosome (Fig. 4).

### Intergenic region detection and differential expression

Regulation of multiple components of the bacterial cell can be performed by the expression of sRNAs. The *trans*-acting sRNAs, in particular, are often effective at regulating multiple different transcripts as opposed to the typically limited scope of *cis*-acting sRNAs. Since *trans*-acting sRNAs are transcribed from IGRs, detection of these sRNAs relies on the accurate identification of IGRs. In order to be classified as a putative sRNA, the region had to adhere to the following characteristics: the differentially expressed region of interest is between two genes, and a significant difference in read depth between the region of interest and the flanking genes exists, so the differential expression does not appear to be a result of flanking gene transcription. If both flanking genes faced the same direction, the read depth was then evaluated. When flanking genes pointed in the same direction and showed similar read depth including the IGR, the region was labeled as an operon structure and eliminated from sRNA candidates. The regions with read depths that were not entirely equivalent to the nearby genes were labeled as possible sRNAs.

For this detection, the entire transcriptome of wild-type HSC5 (WT) and the RNase III null mutant (ΔRNase III) were sequenced. Using the raw RNA-seq data, two differential expression pipelines Cufflinks and RSEM/EBSeq were executed, adding a step after Tophat alignment (Fig. 3). This additional step included a novel script written in Python that identified the RNA transcripts that were transcribed from IGRs (Fig. 2). Cufflinks identified 948 differentially expressed regions, while RSEM/EBSeq discovered 556 regions. The results of both pipelines were combined to find the IGRs that were detected by both programs. Within these IGRs, 376 were identified as having some manner of differential expression between WT and ΔRNase III. In order to select the best potential regions for further testing, those regions with a *p*-value ≤0.05 and an absolute fold change >2 were identified, leaving us with 12 potential regions (Table 1). These regions were then confirmed through visual analysis and rho-independent terminator predictions.

**Table 1:** Detected IGRs with potential for sRNA expression and their confirmation

TSS#^a^	Fold Change (ΔRNase III/HSC5)	*p*-Value	Visual Confirmation using Bamview	Predicted Rho-independent Terminator	sRNA Potential
59	−2.81	5.00E-05	Negative	Yes	Unlikely
72	4.42	5.00E-05	Negative	No	Highly Unlikely
241	2.77	0.0216	Negative	No	Highly Unlikely
231	2.48	0.00095	Negative	Yes	Likely
627	2.22	0.00465	Negative	No	Highly Unlikely
53	2.26	0.0436	Negative	Yes	Unlikely
333	2.37	0.00135	Positive	No	Likely
516	2.24	0.02875	Positive	No	Likely
332	2.29	5.00E-05	Positive	Yes	Highly Likely
795	2.33	0.02665	Positive	No	Likely
181	2.05	0.0059	Positive	No	Likely
520	2.10	0.0405	Positive	Yes	Highly Likely

^a^An arbitrary numeric identifier provided for unannotated region by Cufflinks

### Confirmation of detected regions

**Fig. 5:**
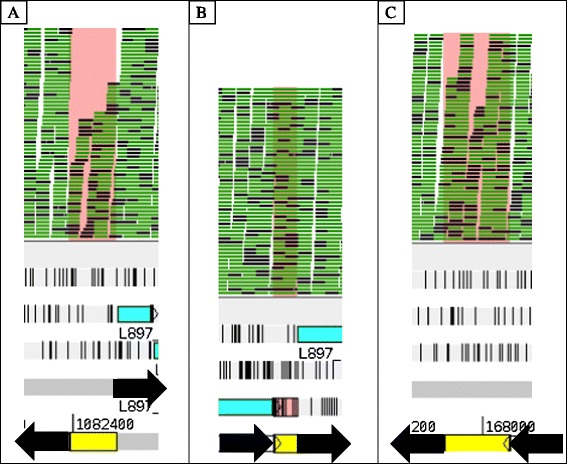
Examples of visual confirmation of sRNAs using Artemis Genome Viewer. Pink highlighted areas represent regions detected by our software and black arrows were added to show flanking gene direction. (**a**) An IGR encoding a putative sRNA, (**b**) An IGR in a predicted operon structure, and (**c**) A IGR encoding a putative sRNA even though the flanking genes are in the same direction

In order to ascertain the validity of the potential regions identified above, we turned to an in silico visual confirmation using the Artemis software provided by the Sanger Institute. Visual confirmation detected regions involved in operon structure or regions that have low enough read support to be determined to be false positives. We proved the final decisions on the 12 identified regions, with 6 of these (~50%) being confirmed as either likely or highly likely sRNA (Table 1). For added clarity, three examples of these visual confirmations are shown in Fig. 4. Operon structures were determined by gene direction and by uniformity of read depth as depicted in Fig. 5b.

For further confirmation, we also looked for predicted rho-independent terminators within the regions that were detected by our software pipeline. The ARNold software uses two different methods for prediction. The top predictions are shown in Table 1. The terminators detected were then confirmed through manual checks. If the predicted terminator was at a location to allow for the termination of putative sRNAs, rather than the flanking gene regions, it was utilized for the ranking of sRNA potential. These predictions were combined with the visual confirmation. Any region with both positive visual validation and a manually validated terminator was considered a highly likely sRNA. If visual validation was positive but no terminator was detected then the region was considered a likely sRNA. A region with only a putative terminator was considered unlikely to be an sRNA while those without any supporting evidence were considered highly unlikely. This analysis left 6 out of the 12 regions to be considered likely sRNAs or better. The detailed region information for these potential sRNAs is given in Table 2.

**Table 2:** The information of IGRs encoding a putative sRNAs that are differentially expressed in ΔRNase III compared to the wild type

TSS #	Left Gene (Direction)	Right Gene (Direction)	Sequence^a^
TSS333	L897_03295 (←)	L897_03295 (→)	ATCTCAGATTAAATTATACCAAAAATGTGAAGCTAATGCTTGTTGTAAGTTCAAATTTAGTAGGATTTTTTATCAGATTTTGTTATAATAAAAACTATGAATAAACTCTATATTGATTCTTTTGTCGAAAAGAAGCTGACAGCAGGGGTACAATTATTAGATGA
TSS516	L897_04905 (→)	L897_04905 (←)	GACTTTCTTTTAAACTATGACACACTATAGTTTAAAAGAAAGTTTTTTTCAGTGTTCATAGTAAATAAAAAAACCGTCTTCCATCAAATAGAAGCGGTTTATCAAATTAACACCAAACCTTAATGCTGTAAGAACCAAGATATAACATCTTTTCCAAAAATAAATAAATAACTGTAGCCGAT
TSS332	L897_03290 (→)	L897_03290 (←)	ACCTAGAAAATAACTTTTTATTACCTATAGAAAGTTATAAAGAAACAAAAATGAAGGAGACGATGGACGTCTTCTTTTTATTATACTCAATATAATAAAAAGAAGTTTCCCATGTTTTATTACCAGTAATGTGGGATATTTAGATGGTAGCAAGAAGTTTTATAGTTGATTTGTTTTCTTTAGGTCTAATTAGCATATTTTGATTACTGATAAACTTGAATAT*CGCCAATAAAAAAGAAGCAAAATTATTATTTTGCTTCTAGTCAGAT*C*A*TTAAATTGATTATTCTCTATTTTTGGTGTTATCCTCTTTTAGTTCAGAAGTTGCCGCGTCAGCTTCTACGGGAT
TSS795	L897_07905 (←)	L897_07905 (→)	CCACACTAAAATGAGATTAGTTAATCATGTTAAGTTTATTAAAAACTTCGGTTTTTATGAAGTCAAGTTTTTAGAGAGTTTTTAGACCATCTTTTACGATACCTTTTGCTTTAACCTCTTTTATGGTATCATATTTTATATAAAGAAAAGGAGAAAAATATGTCCGCCAAGAAAACTTTTTTTGCAAGTAATTTAAAGTACCTTAGATTAAAAAAGAACATGG
TSS181	L897_01895 (→)	L897_01895 (←)	AAGCTCTCGTGTCCCCTATCACATGCATAGGATCAGTGCACTCGACCTTTCAAGACAAGCAAGCATCAGCTCTTGCTTGTCTTTTTTTGGCCTCAAAGCCCGTTAGTCTGCTGCTATGCGAGGCTTTTTTTGAGCATCAGAACGTCAAAAAAAAGGACATGGAGTCCTTTTTTGGTGATCGGTGTTGAGGCCGTCACAAACTGCCCTTGAAATACGCTTCTATGTGGAGCTTTTTTTGGTCCTGTGACACGTAAGCTCC
TSS520	L897_04925 (←)	L897_04925 (←)	CAATTTGCTTAGCAAGTATACTATATTTAAATAATAATTCAACTATAATTTTAAAAAAACACAAAAAAAACATTATACAGCTATAAAGCTTAATATAATAGGATTTTATGTATACAATTATTTAACAGCATCTATTCAAGATCGCCTACTTCATCAGGTTGGTATGACTAAGTTTTTAACTTATCTTCCCCCCTTTTTTTGTTTTAGAAGATAAAAGAATTTTCTTGATTTTGCACACA*AAAAACCGCCCTCAACTAAGAGAGCGGTTGGTTTTTTATTTAA*GGAGACAGTGACT

^a^italics denote predicted terminator regions

The RNase III family of endoribonucleases has been well studied across all organisms, from bacterial RNase III to the Drosha/Dicer family in eukaryotes [37]. Bacterial RNase III typically contains two major regions: the endonuclease domain followed by a double-stranded RNA (dsRNA) binding domain [38]. RNase III is able to regulate gene expression through RNA degradation or binding to target RNAs. RNase III binding helps to stabilize its targets, which can then affect the translation of downstream genes [39–42]. Cleavage of dsRNA by RNase III has been shown to be a major post-transcriptional regulation [43]. Transcripts targeted by RNase III are cleaved into pieces that are average 10–18 bp in length. For a description of the complex mechanics of this process, see Gan et al. but put simply, dsRNA is detected and bound to the dsRNA binding domain, which then cleaved via hydrolysis in the catalytic site of the RNase III homodimer [37, 44]. It is highly plausible that dsRNA formed between an sRNA and its target mRNA could be a substrate of RNase III [45], and our study discovered the putative sRNAs that can be RNase III substrates. The targets and regulatory mechanisms of the putative sRNAs identified from this study could be further studied in the future. A better understanding of sRNA degradation could be used to help study various sRNAs, including those potentially involved in virulence regulation.

## Conclusions

Through the development of an automatic step to add to the typical RNA-seq processing pipeline that detects and isolates the reads that align to IGRs, our study has shown that it is effective in finding potential sRNAs. Through visual validations, we were able to estimate that our methods had a recovery rate of 50% for finding potential sRNAs for the studied samples in *S. pyogenes*.

## Abbreviations

cDNA: Complementary DNA
DNA: Deoxyribonucleic Acid
dsRNA: Double Stranded Ribonucleic Acid
GAS: Group A Streptococcus
GFF: Genome File Format
IGR: Intergenic Regions
mRNA: messenger Ribonucleic Acid
PCR: Polymerase Chain Reaction
RNA: Ribonucleic Acid
RNA-seq: Ribonucleic Acid Sequencing
*S. pyogenes*: *Streptococcus pyogenes*
SAM: Sequence Alignment Map
sRNA: small Noncoding-Ribonucleic Acid
WT: Wild-type
ΔRNase III: Ribonuclease III null *Streptococcus pyogenes* mutant
